# Efficacy and safety of tyrosine kinase inhibitors alone or in combination with radiation therapy for metastatic renal cell carcinoma: A protocol for evidence-based systematic review and Bayesian network meta-analysis

**DOI:** 10.1097/MD.0000000000027004

**Published:** 2021-08-20

**Authors:** Shihua Wang, Weihua Jin, Changqing Jiang, Yiwen Zhang, Kaiwen Deng

**Affiliations:** Department of Pharmacy, The General Hospital of Western Theater Command, Sichuan, China.

**Keywords:** meta, metastatic renal cell carcinoma, protocol, radiation therapy, tyrosine kinase inhibitor

## Abstract

**Background:**

Given the lack of evidence for survival benefit in patients with metastatic renal cell carcinoma from the addition of radiation therapy to tyrosine kinase inhibitor therapy, this Bayesian network meta-analysis aimed to evaluate survival outcomes in patients receiving radiation therapy plus tyrosine kinase inhibitor therapy.

**Methods:**

The preferred reporting items for systematic reviews and meta-analyses reporting guidelines were followed to conduct this study. The electronic databases of PubMed, Cochrane Library, EMBASE, and Web of Science were searched from the inception to August 2021. All phase III clinical trials that reported the outcomes of tyrosine kinase inhibitor with radiation therapy compared with those of tyrosine kinase inhibitor or radiation therapy alone for patients with metastatic renal cell carcinoma were considered eligible for inclusion in this meta-analysis. Overall survival as the primary outcome of interest, and adverse events as secondary outcome of interest were recorded for meta-analysis.

**Results:**

A Bayesian network meta-analysis is an appropriate statistical method to compare all treatment options by statistically simulating the estimated results of a comprehensive trial, and to compare treatments by common and associated comparators. In addition, Bayesian network meta-analysis can produce ranking probabilities of treatments, which may contribute to clinicians’ clinical decision-making.

## 1 Introduction

Renal cell carcinoma is one of the ten most common malignancies in the world, with a steadily increasing incidence. It mainly affects patients 60 years and older. Surgery is the standard treatment for primary renal cell carcinoma. However, approximately 30% of patients with renal cell carcinoma experience local recurrence after surgery, and the other 30% eventually develop metastasis.^[1–3]^ Historically, renal cell carcinoma has been defined as a radiation resistant tumor, especially to conventional doses of radiation therapy, due to the intrinsic resistance of renal cell carcinoma cells to standard doses of radiation.^[4]^ A published characterization of the molecular and genetic characteristics of renal cell carcinoma shows a lack of mutations in genes responsible for DNA repair.^[5]^ This partly explains the resistance of renal cell carcinoma to conventional fractionation and systemic therapy. At the same time, early in vitro cell culture studies have shown that ablative doses of radiation – in which high-dose radiation therapy is administered through a small portion – can effectively eradicate renal cell carcinoma cells.^[6,7]^

New regimens such as tyrosine kinase inhibitors and checkpoint inhibitors have been identified as effective therapies for metastatic renal cell carcinoma, but only a small number of patients achieve complete responses. Additional strategies are needed to improve treatment outcomes. One strategy relies on the combination of tyrosine kinase inhibitor and checkpoint inhibitors with radiation therapy, thus resulting in an increased sensitivity of renal cell carcinoma to the effects of ionizing radiation due to the synergies between these modes.^[6,8]^ This synergistic effect may lead to the development of a distressing effect in which tumor regression is observed in the nonirradiated region, which is assumed to be driven by a cascade of events mediated by the immune system.^[9]^

Current studies in patients with metastatic renal cell carcinoma have predominantly focused on the role of radiation therapy alone in oligometastatic or oligoprogressive settings.^[10–12]^ The safety of radiation therapy combined with tyrosine kinase inhibitors is largely unknown and must be determined by a systematic review. Given the lack of evidence for survival benefit in patients with metastatic renal cell carcinoma from the addition of radiation therapy to tyrosine kinase inhibitor therapy, this Bayesian network meta-analysis aimed to evaluate survival outcomes in patients receiving radiation therapy plus tyrosine kinase inhibitor therapy. A Bayesian network meta-analysis is an appropriate statistical method to compare all treatment options by statistically simulating the estimated results of a comprehensive trial, and to compare treatments by common and associated comparators. In addition, Bayesian network meta-analysis can produce ranking probabilities of treatments, which may contribute to clinicians’ clinical decision-making.

## 2 Materials and methods

### 2.1 Searching strategy

The systematic review protocol was registered on Open Science Framework registries (10.17605/OSF.IO/52498). The preferred reporting items for systematic reviews and meta-analyses reporting guidelines were followed to conduct this study. The electronic databases of PubMed, Cochrane Library, EMBASE, and Web of Science were searched from the inception to August 2021 using the following key terms: “tyrosine kinase inhibitor,” “radiation therapy,” and “metastatic renal cell carcinoma,” for all relevant studies. Furthermore, the reference lists from published original articles and relevant reviews were assessed to identify more relevant studies. Only English publications were included. Ethical approval was not necessary because the present meta-analysis was performed on the basis of previous published studies.

### 2.2 Eligibility criteria

All phase III clinical trials that reported the outcomes of tyrosine kinase inhibitor with radiation therapy compared with those of tyrosine kinase inhibitor or radiation therapy alone for patients with metastatic renal cell carcinoma were considered eligible for inclusion in this meta-analysis. Biomechanical studies, in vitro studies, review articles, techniques, case reports, letters to the editor, and editorials were excluded.

### 2.3 Data extraction

Data were extracted by review of each study for population, mean age, gender, follow-up duration, study design, publishing date, characteristics, and outcomes assessment. Overall survival as the primary outcome of interest, and adverse events as secondary outcome of interest were recorded for meta-analysis. The 2 reviewers created a study-specific spreadsheet in Excel for data collection. Data extraction was performed independently, and any conflict was resolved before final analysis. Any disagreements between the 2 reviewers were discussed and, if necessary, the third author was referred to for arbitration. If the data were missing or could not be extracted directly, authors were contacted by email. If necessary, we would abandon the extraction of incomplete data.

### 2.4 Statistical analysis

We used WinBUGS 1.4.3 software (MRC Biostatistics Unit, Cambridge, UK) and NetMetaXL (Canadian Agency for Drugs and Technologies in Health, Ottawa, Canada) to conduct a Bayesian network meta-analysis. Network meta-analysis combined data from several different randomized comparisons of different treatments to provide an internally consistent set of estimates, while respecting randomization in each trial. The network meta-analysis was performed within a generalized linear model framework with a link function that specified the relationship between the results and the model coefficients to be estimated. When the outcome was continuous, the likelihood was modeled as normal. When the outcome was the event rate, the likelihood was modeled as Poisson. The random effects model was used for this analysis. Estimation was performed in a Bayesian context using the noninformation prior distribution of the parameters. The model was evaluated using the deviation information criterion, a measure that combines model fit and complexity. The analysis was estimated using a Bayesian Markov Chain Monte Carlo model.

### 2.5 Quality evaluation

Each paper was reviewed by 1 reviewer and verified by a second and disagreements were resolved by discussion with a third reviewer. A meta-analysis was conducted when 3 or more trials reported an outcome of interest. Subgroup analyses were planned based on different follow-up periods and the status of the pain assessment. We would also perform the sensitivity analysis to evaluate whether the differences of study design had an impact on the overall estimate and data. The Cochrane risk of bias tool was independently used to evaluate the risk of bias of included randomized controlled trials by 2 reviewers.

## 3 Discussion

Current studies in patients with metastatic renal cell carcinoma have predominantly focused on the role of radiation therapy alone in oligometastatic or oligoprogressive settings.^[10–12]^ The safety of radiation therapy combined with tyrosine kinase inhibitors is largely unknown and must be determined by a systematic review. Given the lack of evidence for survival benefit in patients with metastatic renal cell carcinoma from the addition of radiation therapy to tyrosine kinase inhibitor therapy, this Bayesian network meta-analysis aimed to evaluate survival outcomes in patients receiving radiation therapy plus tyrosine kinase inhibitor therapy. A Bayesian network meta-analysis is an appropriate statistical method to compare all treatment options by statistically simulating the estimated results of a comprehensive trial, and to compare treatments by common and associated comparators. In addition, Bayesian network meta-analysis can produce ranking probabilities of treatments, which may contribute to clinicians’ clinical decision-making.

## Author contributions

**Conceptualization:** Changqing Jiang.

**Data curation:** Shihua Wang, Weihua Jin.

**Formal analysis:** Shihua Wang, Weihua Jin.

**Funding acquisition:** Kaiwen Deng.

**Investigation:** Shihua Wang, Weihua Jin.

**Methodology:** Changqing Jiang.

**Project administration:** Kaiwen Deng.

**Resources:** Kaiwen Deng.

**Software:** Changqing Jiang.

**Supervision:** Yiwen Zhang.

**Validation:** Weihua Jin.

**Visualization:** Weihua Jin.

**Writing – original draft:** Shihua Wang.

**Writing – review & editing:** Yiwen Zhang.
